# A Successful Cross-Agency Model for Collaborative Response to Imported Infectious Disease: Investigation and Management of the First Confirmed Clade Ia Mpox Case in Shanghai, China

**DOI:** 10.3390/tropicalmed11080212

**Published:** 2026-07-28

**Authors:** Zilian Cao, Fang Xu, Xiaoyan Huang, Yifeng Shen, Xin Xin, Lin Ai, Hao Pan, Huanyu Wu

**Affiliations:** 1 Surveillance and Early Warning Institute, Shanghai Municipal Center for Disease Control & Prevention, Shanghai 201107, China; 2 Emergency Management Department, Shanghai Municipal Center for Disease Control & Prevention, Shanghai 201107, China; 3 Center Management, Shanghai Pudong New Area Center for Disease Control & Prevention (Shanghai Pudong New Area Health Supervision Institute), Shanghai 200136, China; 4 Department of Tuberculosis and AIDS Prevention & Control, Shanghai Pudong New Area Center for Disease Control & Prevention (Shanghai Pudong New Area Health Supervision Institute), Shanghai 200136, China; 5 Institute of Pathogen Biology and Testing, Shanghai Municipal Center for Disease Control & Prevention, Shanghai 201107, China; 6 Institute of Infectious Disease Prevention and Control, Shanghai Municipal Center for Disease Control & Prevention, Shanghai 201107, China; 7 Center Management, Shanghai Municipal Center for Disease Control & Prevention, Shanghai 201107, China

**Keywords:** cross-agency collaborative mechanism, clade Ia mpox, imported infectious disease

## Abstract

Introduction: Historically, human infections with clade Ia monkeypox virus (MPXV) primarily occurred through zoonotic transmission with limited secondary human-to-human transmission. Recent studies suggest increasing evidence of human-to-human transmission of some clade Ia lineages. This report documents the first imported clade Ia mpox case in Shanghai, representing the second documented case in China. Methods: Patient demographics, clinical profiles, epidemiological history, and close contacts were obtained through interviews with the patient and attending physicians. Clinical specimens were collected for real-time fluorescent quantitative polymerase chain reaction (qPCR) and whole-genome sequencing. Contact tracing was implemented, involving a 21-day health monitoring of close contacts. Results: The patient presented with moderate symptoms, including fever, rash, and respiratory manifestations. Genome sequencing identified the virus as clade Ia, showing 99.96% nucleotide identity to a strain from the Democratic Republic of Congo (DRC). Eight close contacts were identified with no secondary infection reported. Conclusions: This case was confirmed as an imported clade Ia mpox case originating in the DRC. The “four JOINTs” cross-agency collaborative mechanism between the CDC and customs played a significant role in effectively stopping local transmission. Challenges in remote investigation highlight the need for stronger international collaboration. Establishing an integrated “early warning-control-tracking” system, coupled with public health intervention, is crucial for reducing importation risks and safeguarding national health security.

## 1. Introduction

Mpox is a zoonotic infectious disease caused by the monkeypox virus (MPXV), a member of the Orthopoxvirus genus within the family Poxviridae [1]. MPXV is genetically classified into two major clades: Clade I (subclades Ia and Ib) and Clade II (subclades IIa and IIb) according to standardized WHO viral nomenclature guidance [2]. The global multi-country mpox outbreak spanning 2022–2023 was overwhelmingly driven by Clade IIb strains, which sustained widespread human-to-human transmission among specific populations outside Africa [3]. In contrast, clade Ia strains have long been endemic in Central Africa, mainly the Democratic Republic of Congo (DRC), featuring markedly higher virulence with historical case fatality rates up to 10% compared with Clade IIb infections [4]. Traditionally, clade Ia was presumed to transmit primarily via zoonotic spillover with limited secondary human spread. However, recent studies suggest increasing evidence of human-to-human transmission of some clade Ia lineages [5,6]. As of 31 December 2025, four imported cases of MPXV clade Ia have been documented in three non-endemic countries outside Africa, with one case each confirmed in Ireland and Turkey [3] in addition to China. In April 2025, Shanghai reported its first clade Ia mpox case, representing the second documented case in China. This was a colleague of an imported case from the DRC reported by Shandong Province in March 2025 (Case S) [7]. This report aims to describe the epidemiological investigation, laboratory confirmation, and public health response associated with Shanghai’s first imported clade Ia mpox case. By detailing the comprehensive response measures implemented, including the “four JOINTs” mechanism, this case report offers practical lessons for managing similar imported cases and strengthens global preparedness against emerging MPXV lineages.

## 2. Materials and Methods

Patient demographics, clinical profiles, and epidemiological history were obtained through interviews with the patient and attending physicians. All epidemiological investigation, close contact management, and laboratory testing strictly followed the National Mpox Prevention and Control Protocol of China [8].

### 2.1. Case Definition Criteria

A suspected case was defined as a patient presenting with acute rash accompanied by fever (>37.3 °C) or lymphadenopathy, with a relevant epidemiological history within 21 days before symptom onset. A confirmed case was defined as a suspected patient or a close contact with positive mpox virus nucleic acid detected by quantitative real-time polymerase chain reaction (qPCR) or successful viral isolation [8].

### 2.2. Specimen Collection and Laboratory Detection

Skin lesion swabs, oropharyngeal swabs, and blood samples (both anticoagulated and non-anticoagulated) were collected for subsequent laboratory diagnosis. All specimens were sealed with unique identification labels, temporarily stored at 2–8 °C and delivered to the testing laboratory under cold chain within 1 h after sampling.

Viral DNA was extracted using a commercial nucleic acid extraction kit following the manufacturer’s instructions. Real-time quantitative PCR (qPCR) targeting the MPXV F3L gene was performed using fluorescent PCR kits for universal mpox virus, clade Ia, Clade Ib, and Clade II (Beijing Kinghawk Pharmaceutical Co., Ltd., Beijing, China). The result interpretation criteria were as follows: no Ct value was considered negative, a Ct value ≤ 38 was considered positive, and a Ct value between 38 and 42 required retesting for further verification.

### 2.3. Genome Sequencing and Phylogenetic Analysis

Whole-genome sequencing was performed on the DNBSEQ-G99 platform using an ATOPlex multiplex PCR amplicon sequencing approach. Raw sequencing reads were mapped to the MPXV reference genome (GenBank Accession No. NC_003310.1 and NC_063383.1) using Minimap2 (V2.30)/BWA (V0.7.19). A consensus sequence was generated using FreeBayes and bcftools to evaluate genome coverage and sequence gaps. Nucleotide identity between the imported strain and the reference genome was then calculated using Nextclade and MEGA to determine its epidemiological and evolutionary linkage to the ongoing outbreaks in the DRC.

### 2.4. Close Contact Definition and Monitoring

Close contacts were defined as individuals who had direct contact with the patient’s lesions or contaminated materials, engaged in sexual contact, or experienced prolonged unprotected occupational exposure. Identified contacts were registered for 21 days of self-health monitoring starting from the last day of exposure. Active follow-ups were conducted by local health authorities on days 7, 14, and 21. Any contacts developing mpox-like symptoms (e.g., fever, rash) were immediately referred for qPCR testing.

### 2.5. Ethical Considerations

As this research belongs to routine public health surveillance, written informed consent was not required. All personal identifying information was deleted to protect patient confidentiality.

## 3. Results

### 3.1. Epidemiological Investigation and Clinical Course

On 13 April 2025, a 39-year-old male traveler arriving from the DRC was suspected of mpox by Shanghai Customs during entry at Shanghai Pudong International Airport. The suspicion was triggered by his travel history from the DRC and the observation of visible crusted rashes on his face and limbs. The case was subsequently reported to the Shanghai CDC.

Joint investigations were organized by the Shanghai CDC and local district CDC. The case had been working in Kongo Central Province of the DRC prior to entry. The patient reported traveling with Case S in the same vehicle for approximately five hours on 16 March and returned together on 19 March. Their daily contact was limited to dining together in the same space, and no other forms of exposure were reported. The patient reported no history of contact with wild animals or any sexual contact.

In accordance with the National Mpox Prevention and Control Protocol [8], eight close contacts were identified on the same flight, including 3 passengers seated in front, 3 passengers behind, 1 passenger to the left and 1 passenger to the right of the patient’s seat. All eight close contacts left Shanghai. The Shanghai CDC has sent official letters to the four associated provincial CDCs to conduct trace investigations. The relevant CDCs conducted health inquiries and implemented self-health monitoring. If they develop mpox-like symptoms such as fever, rash, or lymphadenopathy, individuals shall seek medical treatment promptly and truthfully report their epidemiological history. None of them developed mpox-like symptoms during the self-health monitoring period (13 April to 4 May).

**Figure 1:**
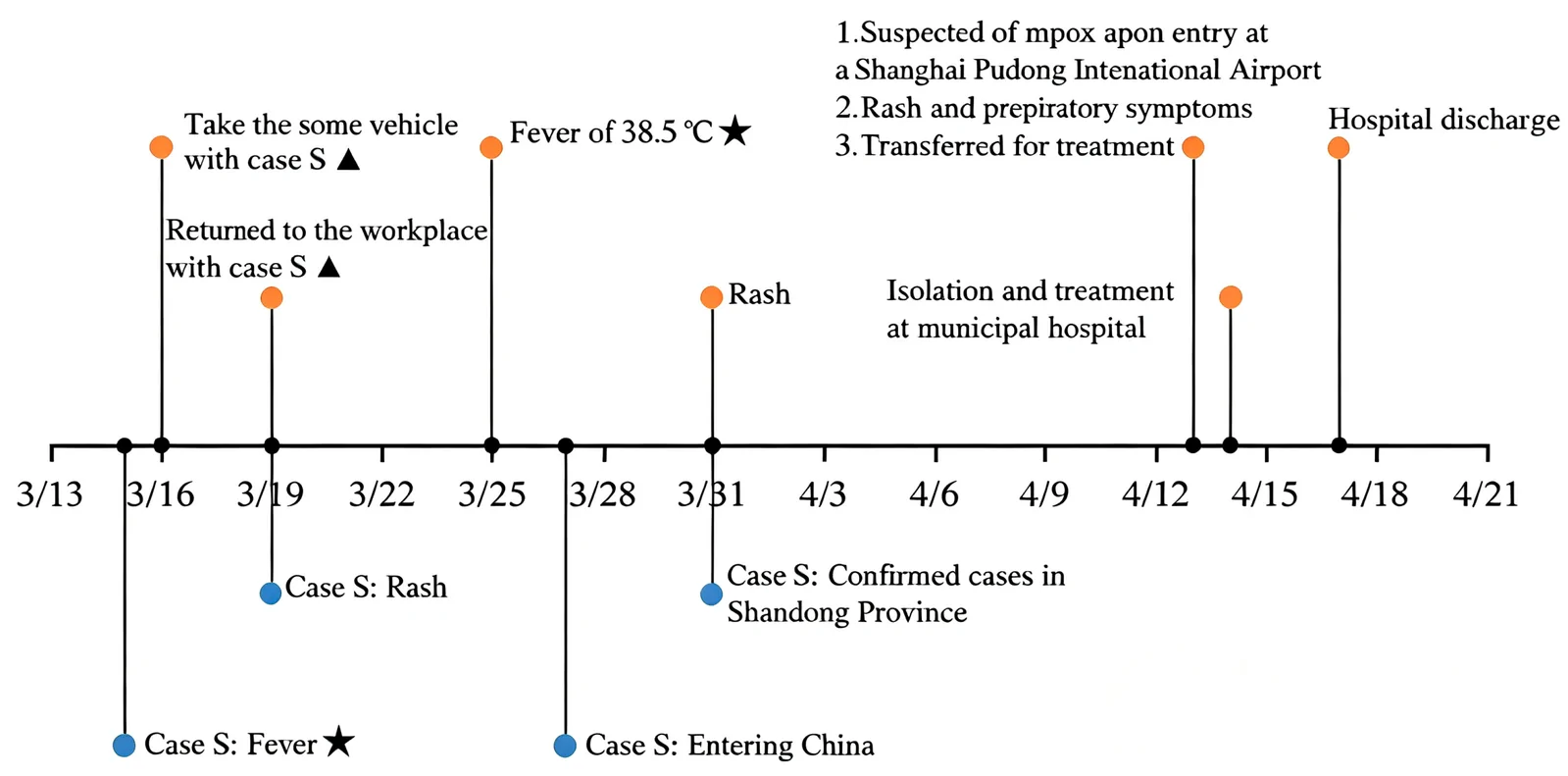
Timeline of Exposure, Onset, and Outcome for the clade Ia Case—Shanghai, 13 April 2025. Triangles indicate suspected exposures; stars indicate onset.

Further inquiry revealed the following clinical course. He developed a fever (38.5 °C) in the DRC on 25 March, followed by skin rashes around the end of March. The case had sought medical advice from the infirmary of his company in Africa and received intravenous ribavirin, Qingkailing, and ganciclovir; however, no definitive diagnosis was made at that time. No fever was observed, but crusted rashes on the face, limbs, and chest were present, and the patient reported a dry cough and sore throat when the case arrived at Shanghai on 13 April. The case was admitted to the hospital with a chief complaint of “fever for 19 days and rash for 14 days.” Physical examination revealed 3–4 scattered crusted lesions (approximately the size of soybeans) on his chest, both arms, and left ring finger; no lymphadenopathy was observed. In addition, two ulcers approximately 1 cm in diameter were found around the anal orifice. The patient was transferred to a municipal-level designated hospital for isolation and treatment on 14 April. After 3 days of treatment, as his lesions had crusted and he met the clinical criteria for home isolation (i.e., clinical symptoms improved and all lesions had crusted), he was discharged to home quarantine on 17 April. The epidemiological timeline, including exposure, onset, clinical consultation, and outcomes, is summarized in Figure 1.

### 3.2. Laboratory Detection and Genome Analysis

Upon entry, testing conducted by Shanghai Customs yielded positive results for MPXV nucleic acid, identifying the genotype as clade Ia. On 13 April, samples were sent to the Pudong CDC. The oropharyngeal swab tested positive, while blood samples were negative. A supplementary vesicular fluid sample collected on 14 April tested positive with a cycle threshold (Ct) value of 27. Further confirmatory testing by the Shanghai CDC showed that the oropharyngeal swab (Ct value: 32) and vesicular fluid sample (Ct value: 25) were positive, with a negative blood sample. The sequencing achieved a valid depth meeting the national requirement (≥10×) and a genome coverage of 99.87%. The consensus sequence belonged to clade Ia and shared 99.96% nucleotide identity with a sequence originating from the DRC in 2024 (GenBank Accession No. OZ254474).

### 3.3. Public Health Response

**Figure 2:**
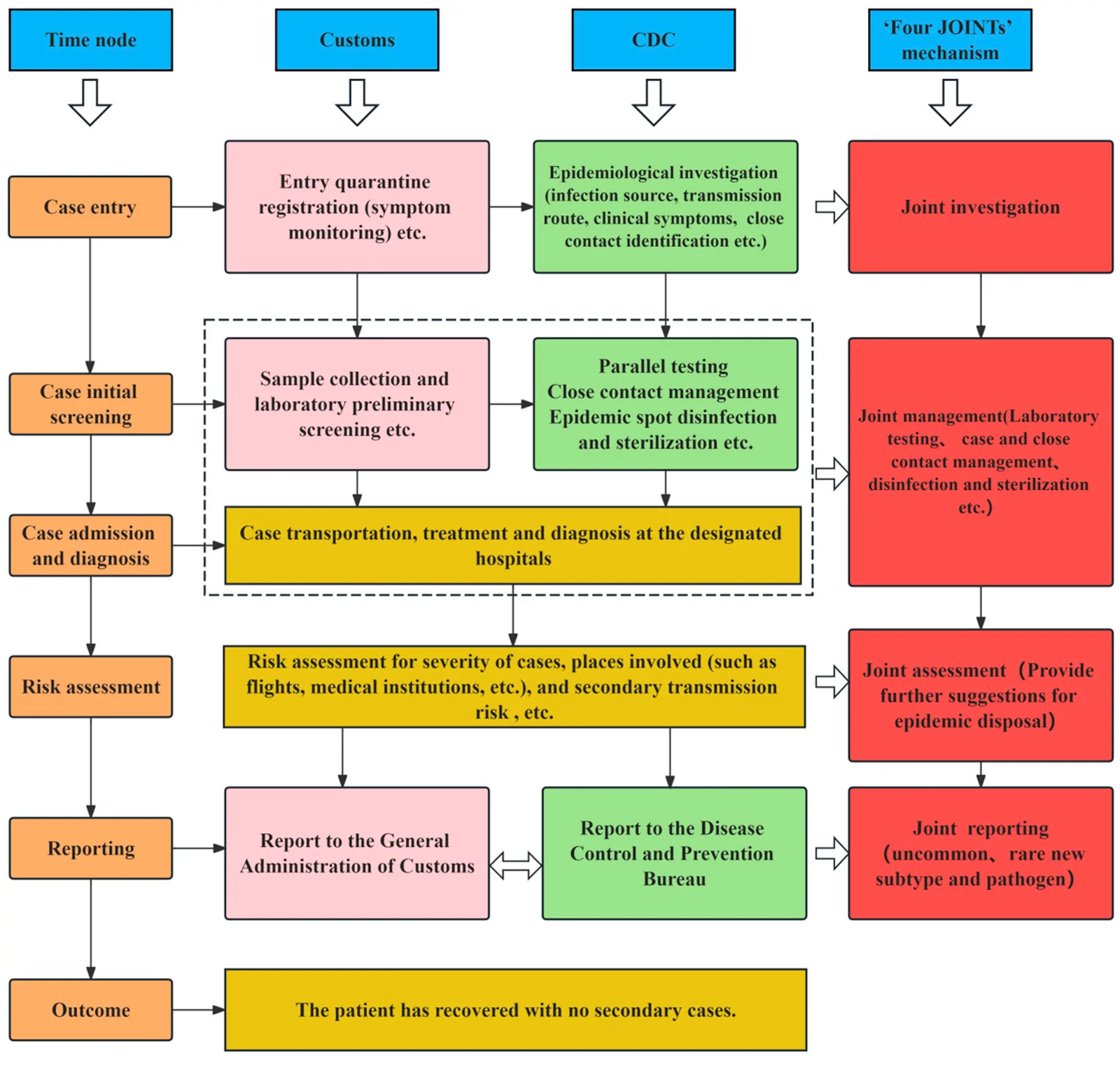
Work Processes and Contents of the “Four JOINTs” Mechanism Between CDCs and Customs.

In 2024, the Shanghai CDC authorities and customs agencies implemented a cross-agency collaborative mechanism encompassing joint investigation, management, assessment, and reporting (“four JOINTs”). Customs is the first gateway for preventing imported epidemics. Suspected cases are detected and registered during customs entry quarantine. After sampling and initial screening of the cases, Shanghai Customs notified the CDC promptly. Upon receiving the notification, the CDC conducted detailed epidemiological investigations, sample reviews, close contact screening, tracing and management. Joint risk assessments were conducted, and case information was reported to the General Administration of Customs and the National Administration of Disease Prevention and Control. The patient recovered after treatment, and no further transmission was detected following these coordinated responses. The specific work contents and processes of both parties under the “four JOINTs” mechanism are detailed in Figure 2.

To prevent transmission, comprehensive containment measures were implemented. The case was transported to designated district and municipal hospitals using dedicated transfer ambulances. Upon admission, the patient was isolated in a single room. All clinical personnel implemented standard precautions in conjunction with contact and droplet precautions. They wore N95 filtering facepieces, isolation gowns, disposable gloves and face shields, and enforced standardized disposal of infectious medical waste. After lesion crusting and clinical improvement, the patient met the national criteria for home isolation and was discharged on 17 April. For close contacts, local CDC staff provided health education emphasizing daily self-monitoring (temperature and rashes), reducing physical contact, and seeking prompt care if symptomatic. Staff conducting on-site follow-ups strictly adhered to personal protection protocols. Additionally, concurrent and terminal disinfection were systematically applied to the patient’s transfer vehicles, medical settings, and activity venues. No secondary cases were detected during the 21-day observation period.

## 4. Discussion

This is the first imported clade Ia mpox case reported in Shanghai and the second in China. The detection of this case underscores the ongoing risk of imported mpox from endemic regions and is of great public health significance. Its value lies not only in the rarity of the case, but also in the successful implementation of the “four JOINTs” cross-agency mechanism. In 2025, mpox was formally incorporated into the list of notifiable infectious diseases under the Frontier Health and Quarantine Law of the People’s Republic of China [9]. This study successfully identified the imported clade Ia mpox case through the “four JOINTs” mechanism. This cross-agency approach provides a practical framework for the prevention and control of imported infectious diseases at international ports.

Clade Ia MPXV predominantly affects endemic areas in Central Africa and is driven by zoonotic transmission, with low secondary human-to-human transmission [3,10]. However, recent genomic and epidemiological studies from the DRC have reported that a new subclade Ia lineage, with APOBEC3 mutations, shows increasing evidence of human-to-human transmission through both sexual and non-sexual close contact (e.g., household and social contact), similar to subclade Ib [5,6]. The case had close contact with Case S on 16 March (9 days before onset) and 19 March (6 days before onset) respectively. On these dates, Case S was on the first and fourth days after illness onset, periods associated with relatively high viral shedding capacity for mpox [11]. Both exposures occurred in closed spaces for a long time, indicating that this contact pattern poses an infection risk. In contrast, when the eight close contacts were exposed on the flight, the case was already on the 19th day after onset, and the rash was mostly scabbed, with no fever resulting in relatively low transmissibility. This also explains the possible reason why no secondary transmission occurred in this cluster. The case denied sexual contact, but the possibility of sexual contact as a route of infection cannot be ruled out without supporting evidence from field epidemiological investigations in Africa.

Although clade Ia has a higher case fatality ratio [4] and severity [11] than the other subclades, the clinical symptoms of this case and the other two previously reported imported clade Ia cases were all relatively mild [7,12]. Regarding clinical manifestations, much remains unknown about the complete clinical phenotype of clade Ia. Historically, rashes associated with this clade have primarily targeted the facial and peripheral limb regions [13], a classic centrifugal feature clearly observed in both the Irish case and our patient [12]. However, a study of clade Ia cases in Kinshasa indicated that genital or anorectal lesions were more common than historically described [6], highlighting the heterogeneity and evolving nature of clinical features. Irrespective of cutaneous lesion distribution, laboratory data corroborated the mild clinical course of this case: high viral loads in vesicular fluid coupled with negative blood samples confirmed localized viral replication without systemic viremia. Viral detection in oropharyngeal swabs further emphasizes the importance of strict respiratory and contact precautions. Whole-genome sequencing identified high nucleotide homology between the isolate and 2024 DRC-circulating clade Ia MPXV strains. Consistent with epidemiological findings, these molecular data confirm the DRC as the importation source and provide critical genomic evidence for tracking cross-border clade Ia MPXV transmission.

To respond to imported infectious diseases, customs authorities have strengthened inspection and quarantine to detect suspicious cases at an early stage. Once a case is identified, they promptly notify the CDC authorities, activate the four JOINTs mechanism, and transfer the patient to a designated hospital for treatment. This collaborative model applies not only to mpox, but also to more than 30 types of infectious diseases quarantined and monitored at points of entry, such as Plague, Cholera, Yellow Fever, Ebola Virus Disease, and Dengue Fever. This mechanism operates effectively through a formalized structure. Both agencies assign dedicated personnel for routine communication; information is shared efficiently through informatization, and risk assessments are conducted regularly or irregularly. Operationally, the success of this containment framework depended on the seamless coordination of multiple infection control interventions. Crucially, healthcare personnel strictly executed dedicated patient transport, single-room isolation, and standardized Personal Protective Equipment (PPE) protocols. These efforts were complemented by rigorous concurrent and terminal disinfection of medical and transport environments, alongside a 21-day surveillance of close contacts. Collectively, these measures effectively eliminated the risk of onward transmission, ultimately resulting in zero secondary cases. The current mechanism lacks support for field epidemiological investigations from overseas. It is necessary to establish a cross-border remote investigation coordination mechanism and promote regular international collaboration among public health authorities. To further strengthen prevention and control of imported infectious diseases and safeguard national health security, it is recommended to issue targeted risk warnings to travelers from affected areas during periods when the WHO issues a Public Health Emergency or during local outbreaks of quarantine and monitored infectious diseases, as well as to facilitate convenient access to medical care for those with abnormal quarantine conditions upon entry. International travelers are also advised to actively report any abnormal symptoms upon entry and to disclose their epidemiological history when seeking medical treatment. These measures will help establish a comprehensive prevention and control system that integrates “remote early warning, entry control, and post-entry case tracing”, thereby significantly reducing the risks of disease importation and local transmission.

## 5. Limitations

One limitation of this study is the lack of continuous monitoring regarding vital signs and the complete clinical evolution of symptoms (such as the daily progression of the rash), thereby restricting a comprehensive understanding of the natural history of clade Ia infections. Furthermore, due to the lack of field epidemiological investigations in the DRC, the possibility of sexual contact as a route of infection could not be definitively ruled out.

## 6. Conclusions

This case was confirmed as an imported clade Ia mpox case originating in the DRC. The “four JOINTs” cross-agency collaborative mechanism between the CDC and customs played a significant role in effectively stopping local transmission. Challenges in remote investigation highlight the need for stronger international collaboration. Establishing an integrated “early warning-control-tracking” system, coupled with public health intervention, is crucial for reducing importation risks and safeguarding national health security.

## Data Availability

The data analyzed in this study involve anonymized epidemiological survey data collected from public health surveillance programs. Restrictions apply to the availability of these data due to relevant privacy and ethical protection regulations for public health survey data. Furthermore, the whole-genome sequence data presented in this study are not publicly available due to institutional and national regulations regarding the biosafety review and management of genomic data for emerging infectious pathogens. The de-identified datasets and sequence information that support the findings of this study are securely stored in the internal database of the Shanghai Municipal Center for Disease Control and Prevention, and are available from the corresponding authors upon reasonable request and with the official approval of the competent health authority.
